# Breast cancer mortality rates are levelling off or beginning to decline in many western countries: analysis of time trends, age-cohort and age-period models of breast cancer mortality in 20 countries.

**DOI:** 10.1038/bjc.1996.171

**Published:** 1996-04

**Authors:** C. Hermon, V. Beral

**Affiliations:** Imperial Cancer Research Fund, Cancer Epidemiology Unit, Radcliffe Infirmary, Oxford, UK.

## Abstract

Age-standardised mortality rates for breast cancer were examined for 20 countries in Europe, North America, Australia and New Zealand from 1950 to 1992 and age-birth cohort and age-period of death models were fitted to the data. Breast cancer mortality rates generally increased in the earlier decades, but more recently rates have levelled off or begun to decline in most countries. Only in 4 of the 20 countries studied, Belgium, Hungary, Poland and Spain, was there no evidence of a decline or leveling off or mortality in recent birth cohorts or in recent years. In the other countries the decline in mortality appeared to be in part due to birth cohort effects and in part due to period effects. The birth cohort effects were suggestive of a decline in breast cancer rates among women born after about 1920 and were evident in many countries especially Canada, The Netherlands, The United Kingdom and the United States. The decline in mortality in women born after 1920 appeared to be in part related to a reduction in childlessness and a reduction in age at first birth in those generations. As well as the birth cohort effects, there was some evidence of a recent overall decline in mortality rates in several countries, e.g. Austria, FRG, Greece and the UK, and this may be due to an increase in survival resulting from improved management and treatment of women with breast cancer.


British Journal of Cancer (1996) 73, 955-960
© 1996 Stockton Press All rights reserved 0007-0920/96 $12.00

### Breast cancer mortality rates are levelling off or beginning to decline in many western countries: analysis of time trends, age–cohort and age–period models of breast cancer mortality in 20 countries

C Hermon[1] and V Beral[1]

[1]*Imperial Cancer Research Fund, Cancer Epidemiology Unit, Gibson Building, Radcliffe Infirmary, Oxford OX2 6HE, UK.*

**Summary** Age–standardised mortality rates for breast cancer were examined for 20 countries in Europe, North America, Australia and New Zealand from 1950 to 1992 and age–birth cohort and age–period of death models were fitted to the data. Breast cancer mortality rates generally increased in the earlier decades, but more recently rates have levelled off or begun to decline in most countries. Only in 4 of the 20 countries studied, Belgium, Hungary, Poland and Spain, was there no evidence of a decline or levelling off of mortality in recent birth cohorts or in recent years. In the other countries the decline in mortality appeared to be in part due to birth cohort effects and in part due to period effects. The birth cohort effects were suggestive of a decline in breast cancer rates among women born after about 1920 and were evident in many countries especially Canada, The Netherlands, The United Kingdom and the United States. The decline in mortality in women born after 1920 appeared to be in part related to a reduction in childlessness and a reduction in age at first birth in those generations. As well as the birth cohort effects, there was some evidence of a recent overall decline in mortality rates in several countries, e.g. Austria, FRG, Greece and the UK, and this may be due to an increase in survival resulting from improved management and treatment of women with breast cancer.

**Keywords:** breast cancer mortality; international comparisons; age standardisation; age–birth cohort and age–period models

Although mortality from breast cancer has generally been increasing worldwide, there have been recent reports of declining mortality in the UK, USA, Norway, Sweden and elsewhere (Blot *et al.*, 1987; Tarone and Chu, 1992; Coleman *et al.*, 1993; Ursin *et al.*, 1994; Beral *et al.*, 1995, 1996).

In this paper we report on mortality trends in different cohorts of women and different periods of time for 20 countries in Europe, North America, Australia and New Zealand.

### Methods

Information on age–specific numbers of deaths from breast cancer and population data for 20 countries were obtained from the World Health Organization's Division of Epidemiological Surveillance and Health Situation and Trend Assessment. Data were provided for each year during the period 1950–92 in 5 year age groups. The countries were selected on the basis that data were available for at least the period 1960–90.

During the period 1950–92 the coding of cause of death spanned the sixth to the ninth revision of the International Classification of Disease (ICD). Breast cancer mortality data used for this analysis were coded using the Basic Tabulation list of the ICD, according to the following codes: [ICD 6 and 7: A051 (WHO, 1949; WHO, 1957), ICD 8: A054 (WHO, 1967), ICD 9: B113 (WHO, 1977)].

Data were initially displayed using mortality rates at ages 30–79 age standardised against the world standard (Parkin *et al.*, 1992) for the individual years that were available. Age, cohort of birth and period of death are not statistically independent, because when two are defined the third is automatically implied and attempts to model all three factors can lead to over parameterisation (Breslow and Day, 1987).

In these analyses two separate models were derived for each country: an age–birth cohort and an age–period of death model. Log-linear Poisson models (Clayton and Schifflers, 1987) were constructed using the statistical package EGRET (Statistics and Epidemiology Research Corporation, 1985).

For the birth cohort models 16 birth cohorts were derived based on year and age at death (e.g. women aged 30–34 who died in 1955–59 belong to the 1920–30 birth cohort). Each birth cohort spans a 10-year interval and is subsequently described by its central year (e.g. 1925). The earliest birth cohorts only include women in the oldest age groups whereas the most recent birth cohorts only include women in the youngest age groups. For the age–period of death modelling eight 5 year calendar periods were used from 1950–89, plus for the most recent period (1990–92) a 3 year period was used. For a few countries data were only available up to 1991.

In fitting the data, the arbitrary reference category used for the age–birth cohort model was the 1920 birth cohort whereas for the age–period of death model the 1950–54 calendar period was used. The parameter estimates produced by the age–cohort modelling are cohort-specific mortality ratios while the estimates produced by the age–period modelling are period-specific mortality ratios. Both sets of ratios are comparable to a relative risk estimate and can be interpreted in a similar manner.

### Results

### *Mortality trends*

Age-standardised mortality data are displayed for the 20 countries in alphabetical order in Figure 1. All rates are standardised to the same population and so the rates are directly comparable. In most countries there has been an overall increase in rates over the first four decades for which data were presented. However, more recently in some countries the rates have levelled off or decreased, e.g. Austria, FRG, The Netherlands and the UK. For some countries the rates have been relatively constant throughout, including Australia, Canada and the USA.

Correspondence: C Hermon
Received 3 August 1995; revised 30 October 1995; accepted 1 November 1995

*Cohort patterns*

The results of age–cohort modelling of the mortality data for different countries are presented alphabetically in Figure 2. Until the recent birth cohorts mortality increased in successive generations of women in most countries. In several countries, e.g. Canada, The Netherlands, Switzerland, the UK and the USA, there has been a downturn in cohort mortality ratios for recent birth cohorts. The downturn started at around the 1920 birth cohort for most of these countries and the mortality ratios have continued to fall in subsequent birth cohorts. Sweden has also shown a downward trend in cohort mortality ratios which has been apparent since the 1910 birth cohort.

Australia has experienced stable cohort mortality ratios for all birth cohorts examined. In France and Italy the cohort

**Figure 1** Annual breast cancer mortality rates per 100 000 women aged 30–79 from 1950 to 1992 in 20 countries in Europe, North America, Australia and New Zealand [standardised by age to the world standard population (Parkin *et al.*, 1992)].

mortality ratios increased markedly for cohorts born up to about 1920 but the rate of increase has slowed down since then. For other countries, e.g. Greece, Hungary and Poland, the cohort mortality ratios have generally been increasing throughout the study period. The cohort patterns for Denmark, Finland, New Zealand and Norway are difficult to interpret, as the ratios fluctuate due to the small populations of these countries (each has a female population of less than 3 million). Nevertheless, Denmark and Norway show some evidence of a downturn in cohort mortality ratios amongst recent birth cohorts.

*Period trends*

The results from the age-period of death modelling are shown in Figure 3. Several countries, e.g. Austria, Canada, Greece, Sweden and the UK, show a levelling off or decline of ratios in recent years where previously they were increasing.

Several other countries, e.g. Australia, The Netherlands, Switzerland and the USA, have experienced fairly uniform ratios throughout the period. For some other countries, e.g. Belgium, France, Hungary, Italy, Poland and Spain, the

**Figure 2** Cohort-specific mortality ratios of breast cancer mortality for women aged 30–79 for cohorts with a central year of birth from 1875 to 1945 (1920 = 1.00) in 20 countries in Europe, North America, Australia and New Zealand.

period mortality ratios are increasing. Trends for Denmark, Finland, Norway and New Zealand are difficult to assess because of small numbers but rates appear to have remained steady or declined.

*Changes in reproductive factors*

To explain the observed patterns in breast cancer mortality in different birth cohorts of women, data on reproductive factors in successive generations of women were sought, but only limited data were available (Council of Europe, 1990; Coleman *et al.*, 1993). Table I shows the percentage of women childless by age 40 for cohorts born in 1930, 1935, 1940 and 1945 for 11 countries. Data for earlier birth cohorts and for other countries were not available. Of the countries which showed a recent fall in breast cancer mortality for cohorts born after around 1920, data could be found for England and Wales, The Netherlands, Norway, Sweden and Switzerland, and all except Switzerland experienced a downward trend in the percentage of women who were childless by age 40 for cohorts born after 1930. However, in two countries which showed no fall in cohort mortality ratios, Belgium and Spain, the percentage of women childless had also decreased. Only in one country (FRG) has there

**Figure 3** Period-specific mortality ratios of breast cancer mortality for women aged 30–79 for years of death 1950–54 to 1990–92 (1950–54 = 1.00) in 20 countries in Europe, North America, Australia and New Zealand.

been an increase in the percentage of women childless by age 40 in recent cohorts, and in that country there was no recent downturn trend in birth cohort mortality ratios.

Data on mean age at first childbirth are presented in Table II. Unfortunately, this type of data was available for only six countries and for cohorts born in 1930 or later. These six countries all show a decline in age at first childbirth amongst women born from 1930 to 1945. For some countries (England and Wales, The Netherlands and Switzerland) the decrease in mean age at first childbirth parallels the fall in cohort-specific breast cancer rates. In the remaining three countries (Austria, France and FRG), while age at first birth is declining there is no parallel fall in cohort breast cancer mortality.

Table III shows the completed family size data for the 1930 to 1945 birth cohorts. All the countries that in our analyses showed a recent downturn in birth cohort mortality ratios also experienced a reduction in the completed cohort fertility for the same birth cohorts.

**Table 1** Percentage of women childless by age 40 by birth cohort (Coleman *et al.*, 1993)

| | Mid-year of birth | | | |
|---|---|---|---|---|
| *Country* | *1930* | *1935* | *1940* | *1945* |
| Austria | 17.2 | 14.8 | 14.3 | 15.1 |
| Belgium | 16.8 | 14.8 | 13.1 | 12.8 |
| England and Wales | 13.8 | 11.4 | 11.1 | 10.2 |
| France | 13.0 | 10.5 | 8.3 | 8.1 |
| FRG | ND | 9.2 | 10.6 | 12.7 |
| Italy | ND | 15.5 | 12.6 | 11.6 |
| Netherlands | 15.4 | 11.7 | 11.9 | 11.7 |
| Norway | ND | 9.6 | 9.5 | 9.2 |
| Spain[a] | 14.2 | 12.0 | 11.0 | 10.0 |
| Sweden | 14.7 | 13.4 | 13.2 | 12.9 |
| Switzerland | ND | 18.6 | 13.9 | 17.6 |

[a]Data were available for the following birth cohorts 1933, 1938, 1943 and 1948. ND, no data.

**Table II** Mean age at first childbirth within wedlock by birth cohort (Council of Europe, 1990)

| | Mid-year of birth | | | |
|---|---|---|---|---|
| *Country* | *1930* | *1935* | *1940* | *1945* |
| Austria | 25.5 | 24.8 | 24.2 | 23.7 |
| England and Wales | 25.2 | 24.7 | 24.1 | 24.2 |
| France | 24.8 | 24.8 | 24.4 | 24.1 |
| FRG | 25.5 | 25.2 | 24.6 | 24.0 |
| Netherlands | 26.0 | 25.5 | 25.0 | 24.5 |
| Switzerland | 26.5 | 26.0 | 25.3 | 25.2 |

**Table III** Completed family size (Council of Europe, 1990)

| | Mid-year of birth | | | |
|---|---|---|---|---|
| *Country* | *1930* | *1935* | *1940* | *1945* |
| Austria | 2.32 | 2.45 | 2.13 | 1.94 |
| Belgium | 2.29 | 2.27 | 2.17 | 1.94 |
| Canada[a] | 3.37 | 3.16 | 2.72 | ND |
| Denmark | 2.36 | 2.38 | 2.24 | 2.06 |
| England and Wales | 2.35 | 2.41 | 2.36 | 2.17 |
| France | 2.64 | 2.58 | 2.41 | 2.22 |
| FRG | 2.14 | 2.17 | 1.97 | 1.77 |
| Greece[b] | 2.21 | 2.02 | 2.01 | 2.00 |
| Italy | 2.30 | 2.28 | 2.15 | 2.08 |
| Netherlands | 2.67 | 2.49 | 2.22 | 1.99 |
| Norway | 2.49 | 2.57 | 2.45 | 2.21 |
| Spain | 2.59 | 2.67 | 2.59 | 2.43 |
| Sweden | 2.11 | 2.14 | 2.05 | 1.96 |
| Switzerland | 2.18 | 2.19 | 2.09 | 1.85 |
| United States[a] | 3.06 | 3.06 | 2.68 | ND |

[a] Data were provided by Coleman. [b]Data were available for the following birth cohorts 1931, 1936, 1941 and 1946. ND, no data.

### Discussion

The main finding from this analysis is that, although breast cancer rates had been increasing in most Western countries since 1950, recently mortality rates seem to have levelled off or begun to decline in many of them. In Australia, Austria, Canada, FRG, Greece, The Netherlands, Sweden, Switzerland, the USA and the UK mortality rates are either constant or have recently levelled off or have begun to decline. The decline appears to be in part due to birth cohort effects and in part due to period effects although it is sometimes difficult to separate these effects using mathematical models. The cohort effects are strongest in Canada, The Netherlands, Sweden, Switzerland, the UK and the USA where cohort mortality ratios have declined for women born after about 1920. In France and Italy breast cancer mortality rates are still increasing, but the rate of increase has slowed down for cohorts born since around 1920. Only in Belgium, Hungary, Poland and Spain have the rates continued to increase throughout the period studied and for all birth cohorts. There is suggestive evidence of a decline in Denmark, Finland, Norway and New Zealand but the population of each country is small and the rates are unstable. These results extend and support the conclusions of others, based on data from earlier years, that breast cancer mortality rates may be declining in many Western countries (Blot *et al.*, 1987; Coleman *et al.*, 1993; Beral *et al.*, 1996).

Several factors might influence the breast cancer mortality rates: change in death certification coding practices; change in incidence rates due to changes in risk factors; and changes in survival due to improvements in treatment and/or earlier diagnoses. Apparent increases or decreases in breast cancer mortality can result from changes in death certification. In all countries included here diseases are classified according to the World Health Organization's International Classification of Disease (ICD) which has not changed for breast cancer over the years of study. In three countries there have been some minor changes in the interpretation of the WHO rules, which mainly affect older women. In Britain, the interpretation of the ICD coding rule was revised in 1984, which resulted in an artefactual increase in breast cancer mortality, mostly in the oldest age groups (OPCS, 1984). In Sweden, additional rules were instituted by the National Central Bureau of Statistics (1983) in 1981 for cause of death coding which produced an artificial lowering of mortality, the effect being strongest amongst the older age groups. In Denmark changes in coding were introduced in 1966 when information from autopsies was included on the death certificate (Ewertz and Carstensen, 1988), consequently, a decline in breast cancer mortality occurred in the oldest age groups due to other more common causes of death being coded. We are unaware of any other changes in cause of death coding that may affect these results. In limiting our analyses to women aged 30–79 the effect of these changes in coding practices should be minimised.

Changes in cohort mortality ratios may be in part due to changes in childbearing patterns (childlessness, parity, age at first full-term pregnancy) for different birth cohorts of women. Blot *et al.* (1987) and Tarone and Chu (1992) reported on the decrease in US breast cancer risk with successive birth cohorts beginning around 1925 and demonstrated how age-specific breast cancer mortality rates parallel changes in childbearing practices, e.g. for women aged 40–59, the decline in breast cancer mortality has been linked to increased fertility during the post-war years. This also appears to have happened in the UK with the average age at first birth and proportion of women childless declining after the second world war (Beral *et al.*, 1996).

It was not possible to obtain data on reproductive factors for all the countries described here. In countries for which data are available there has been a general reduction in the percentage of women childless by age 40 and mean age at first birth between the 1930 and 1945 birth cohorts, and in these countries mortality rates have levelled off or begun to decrease. The only clear exceptions are Belgium and Spain where the percentage of women childless has declined for cohorts born after 1930 but breast cancer rates are increasing. In France and Italy the results are equivocal as the percentage of childless women and the mean age at first childbirth in France has declined (data for Italy were not available) and yet breast cancer mortality rates are still rising, although less rapidly than before. There appears to be little correlation between cohort-specific breast cancer ratios and average family size. This is possibly because ever having had a child and an early age at first childbirth are stronger risk factors for breast cancer than total number of children (Kelsey *et al.*, 1993).

Countries which have shown a recent downturn in mortality are generally the ones which have the highest breast cancer mortality rates, e.g. The Netherlands and the UK, whereas those countries with the lowest breast cancer mortality rates tend to be the ones in which mortality has been increasing, e.g. Poland and Spain. It is possible that what is occurring is a converging of rates to a more common level. This may reflect an international convergence of fertility levels. The prevalence of past oral contraceptive use is increasing among generations of women born since 1930 in Sweden, the UK and the USA (Beral *et al.*, 1996) and in many other Western countries and it is in those birth cohorts that breast cancer rates are declining.

Improvements in survival can also affect mortality trends. The EUROCARE Study has reported on cancer survival in Europe (Berrino *et al.*, 1995). Data are presented for breast cancer for 10 of the 16 European countries included in these analyses. A slight improvement or no change in relative 5 year survival for the period 1983–85 compared with 1978–80 was apparent for nine countries (Denmark, England, Finland, France, FRG, Italy, The Netherlands, Poland and Switzerland). In all except Poland the improvement in survival has been accompanied by stabilising or a decline in mortality rates. In Spain there has been no improvement in relative survival reported by Berrino *et al.* (1995) and this supports the data presented here of increasing mortality during the study period.

Increased survival can be achieved by early detection of tumours and effective treatment. Blot *et al.* (1987) concluded that for women aged less than 40, recent changes in the detection and management of breast cancer have contributed to a reduced mortality in young women in the United States. National policies to promote mammography screening have been established over the last two decades in some countries, although in most countries national screening programmes have only recently been introduced and therefore it is too early for any effect to be seen in mortality rates. Widespread use of adjuvant chemotherapy during the 1980s has been shown to be an effective treatment for breast cancer (Early Breast Cancer Trialists Collaborative Group, 1992). It would seem that early effective treatment may be playing a part in the recent decline in mortality in England and Wales (Beral *et al.*, 1995; Baum, 1995) and perhaps elsewhere; but the effects of such treatment are very recent.

**Acknowledgement**
Mortality data were provided by the World Health Organization.

### References

BAUM M. (1995). Screening for breast cancer, time to think - and stop? (letter). *Lancet*, **346**, 436–437.
BERAL V, HERMON C, REEVES G AND PETO R. (1995). Sudden fall in breast cancer death rates in England and Wales (letter). *Lancet*, **345**, 1642–1643.
BERAL V, HERMON C, REEVES G AND KEY T. (1996). Breast cancer trends in women in Sweden, the UK and the USA in relation to their past use of oral contraceptives. In *Proceedings of the Second International Symposium on Hormonal Carcinogenesis*. Li JJ, Li SA, Nandi A and Gustafsson JA (eds). Springer: Berlin.
BERRINO F, SANT M, VERDECCHIA A, CAPOCACCIA R, HAKULINEN T AND ESTEVE J. (1995). *Survival of Cancer Patients in Europe - The EUROCARE Study*. Scientific Publications No. 132. IARC: Lyon.
BLOT WJ, DEVESA SS AND FRAUMENI JF. (1987). Declining breast cancer mortality among young American women. *J. Natl Cancer Inst.*, **78**, 451–454.
BRESLOW NE AND DAY NE. (1987). *Statistical Methods in Cancer Research*. Vol II. *The Design and Analysis of Cohort Studies*. Scientific Publications No. 82. IARC: Lyon.
CLAYTON D AND SCHIFFLERS E. (1987). Models for temporal variation in cancer rates. I: Age-period and age-cohort models. *Stats. in Med.*, **6**, 449–467.
COLEMAN D. (1993). Britain in Europe: International and regional comparisons of fertility levels and trends. In *New Perspectives on Fertility in Britain*. Ni Bhrolchain M (ed). Studies on Medical and Population Subjects, No. 55. HMSO: London.
COLEMAN MP, ESTEVE J, DAMIECKI P, ARSLAN A AND RENARD H. (1993). *Trends in Cancer Incidence and Mortality*. Scientific Publications No. 121. IARC: Lyon.
COUNCIL OF EUROPE. (1990). *Cohort Fertility in Member States of the Council of Europe*. Population Studies No. 21. Council of Europe Press: Strasbourg.
EARLY BREAST CANCER TRIALISTS' COLLABORATIVE GROUP. (1992). Systemic treatment of early breast cancer by hormonal, cytotoxic, or immune therapy. *Lancet*, **339**, 1–15.
EWERTZ M AND CARSTENSEN B. (1988). Trends in breast cancer incidence and mortality in Denmark, 1943–82. *Int. J. Cancer*, **41**, 46–51.
KELSEY JL, GAMMON MD AND JOHN EM. (1993). Reproductive factors and breast cancer. *Epid. Rev.*, **15**, 36–47.
NATIONAL CENTRAL BUREAU OF STATISTICS. (1983). *Dodsorsaker 1981 - Official Statistics of Sweden*. S-11581. Statistics Sweden: Stockholm.
OFFICE OF POPULATION CENSUSES AND SURVEYS. (1984). *Mortality Statistics – Cause*. Series DH2 No. 11. HMSO: London.
PARKIN DM, MUIR CS, WHELAN SL, GAO YT, FERLAY J AND POWELL J (eds). (1992). *Cancer Incidence in Five Continents*. Vol IV, Scientific Publications No 120. IARC: Lyon.
STATISTICS AND EPIDEMIOLOGY RESEARCH CORPORATION. (1985). *EGRET*. Statistics and Epidemiology Research Corporation: Seattle.
TARONE RE AND CHU KC. (1992). Implications of birth cohort patterns in interpreting trends in breast cancer rates. *J. Natl Cancer Inst.*, **84**, 1402–1410.
URSIN G, BERNSTEIN L AND PIKE MC. (1994). Breast Cancer. *Cancer Surveys*, **19/20**, 241–264.
WORLD HEALTH ORGANIZATION. (1949). *Manual of the International Statistical Classification of Diseases, Injuries and Causes of Death*. WHO: Geneva.
WORLD HEALTH ORGANIZATION. (1957). *Manual of the International Statistical Classification of Diseases, Injuries and Causes of Death*. WHO: Geneva.
WORLD HEALTH ORGANIZATION. (1967). *Manual of the International Statistical Classification of Diseases, Injuries and Causes of Death*. WHO: Geneva.
WORLD HEALTH ORGANIZATION. (1977). *Manual of the International Statistical Classification of Diseases, Injuries and Causes of Death*. WHO: Geneva.


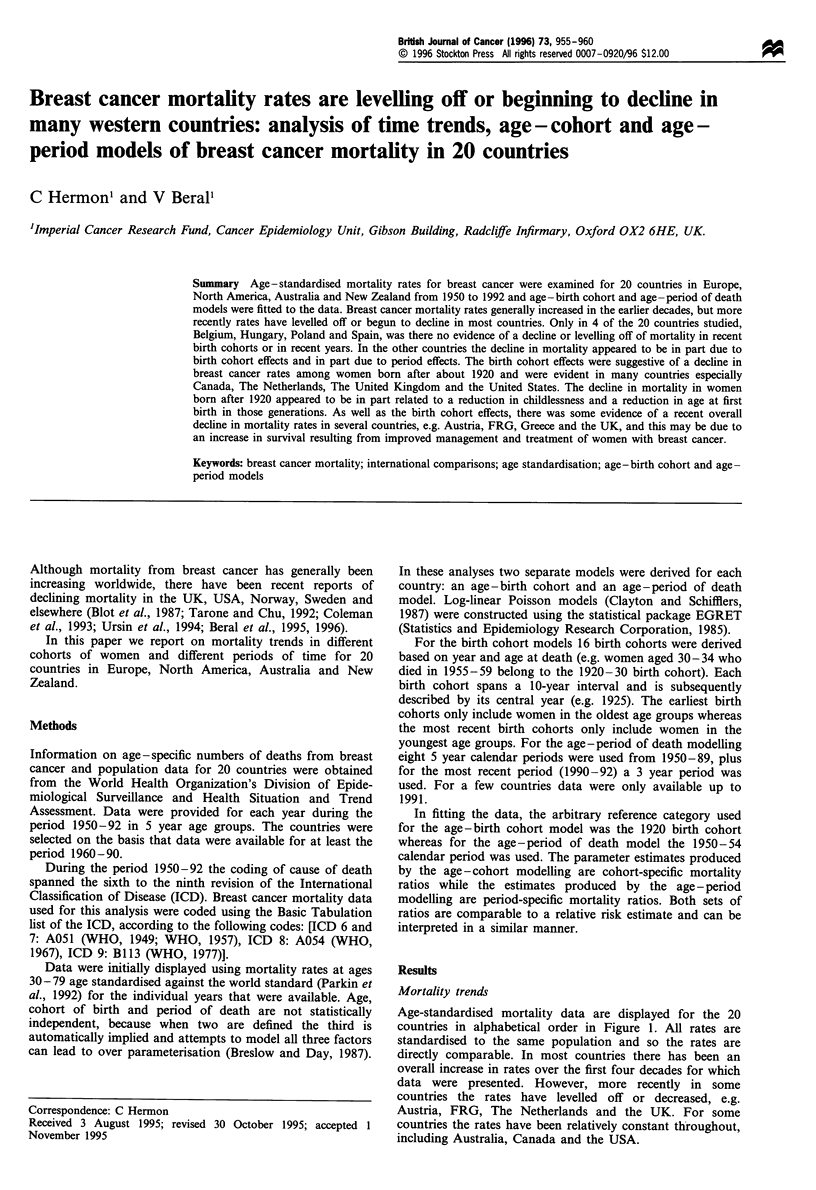

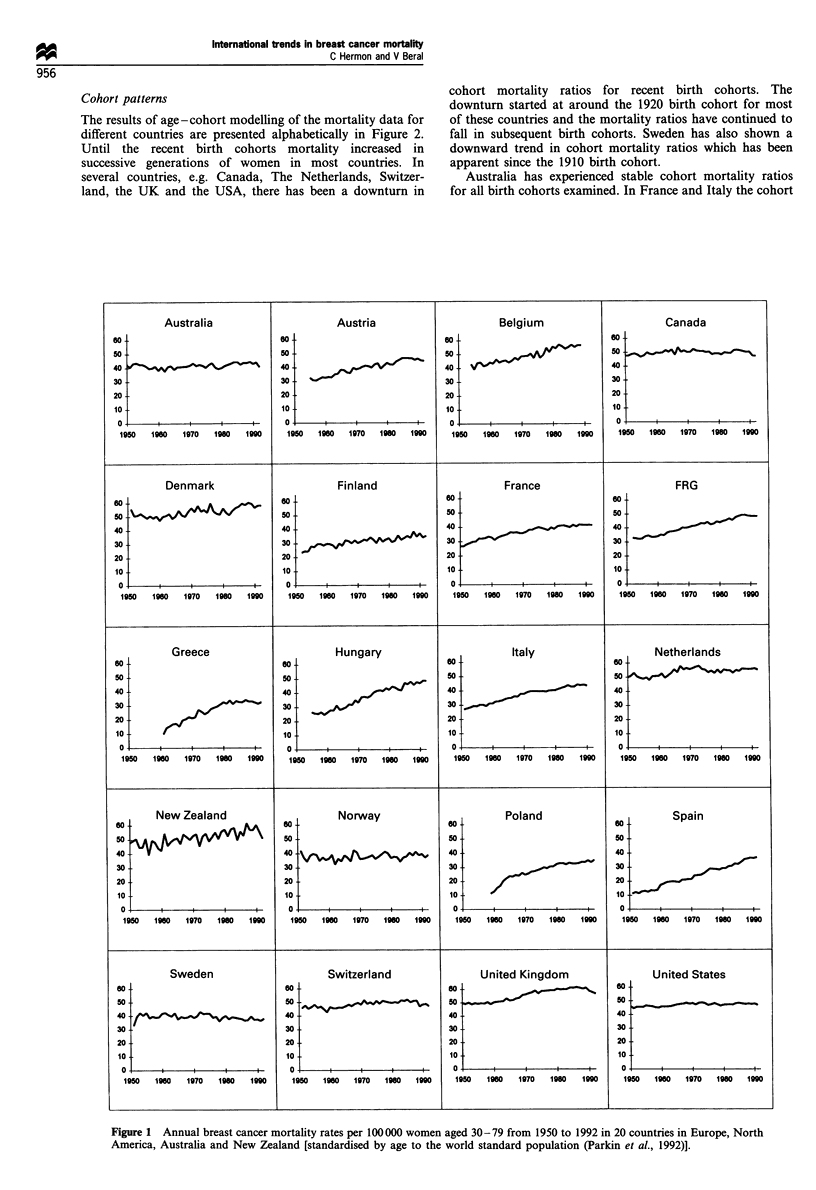

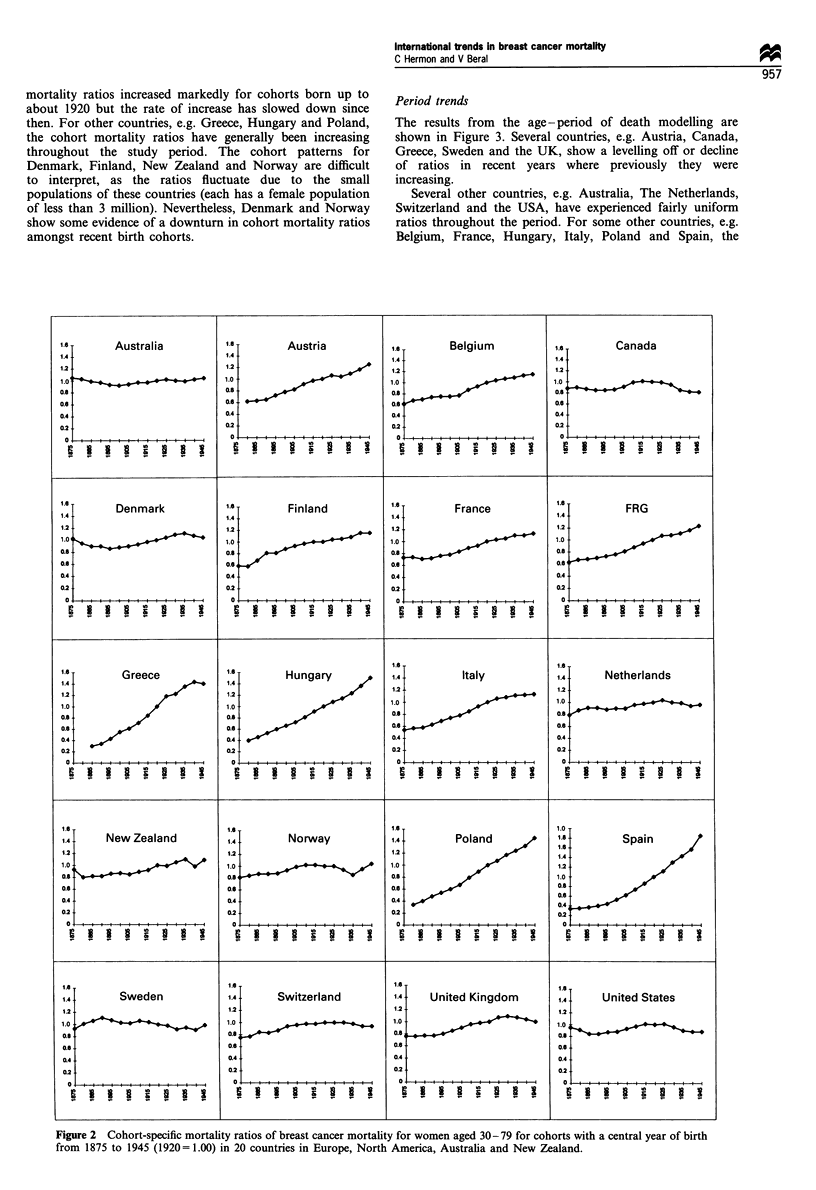

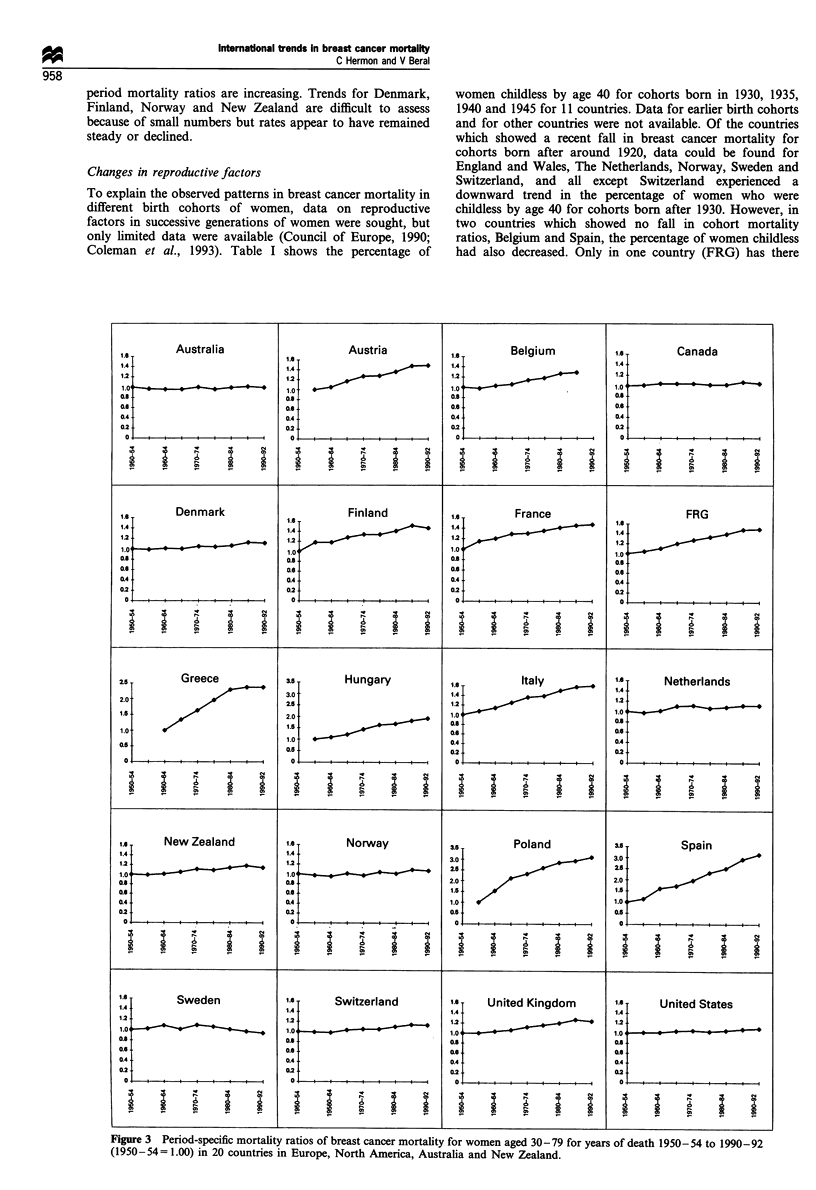

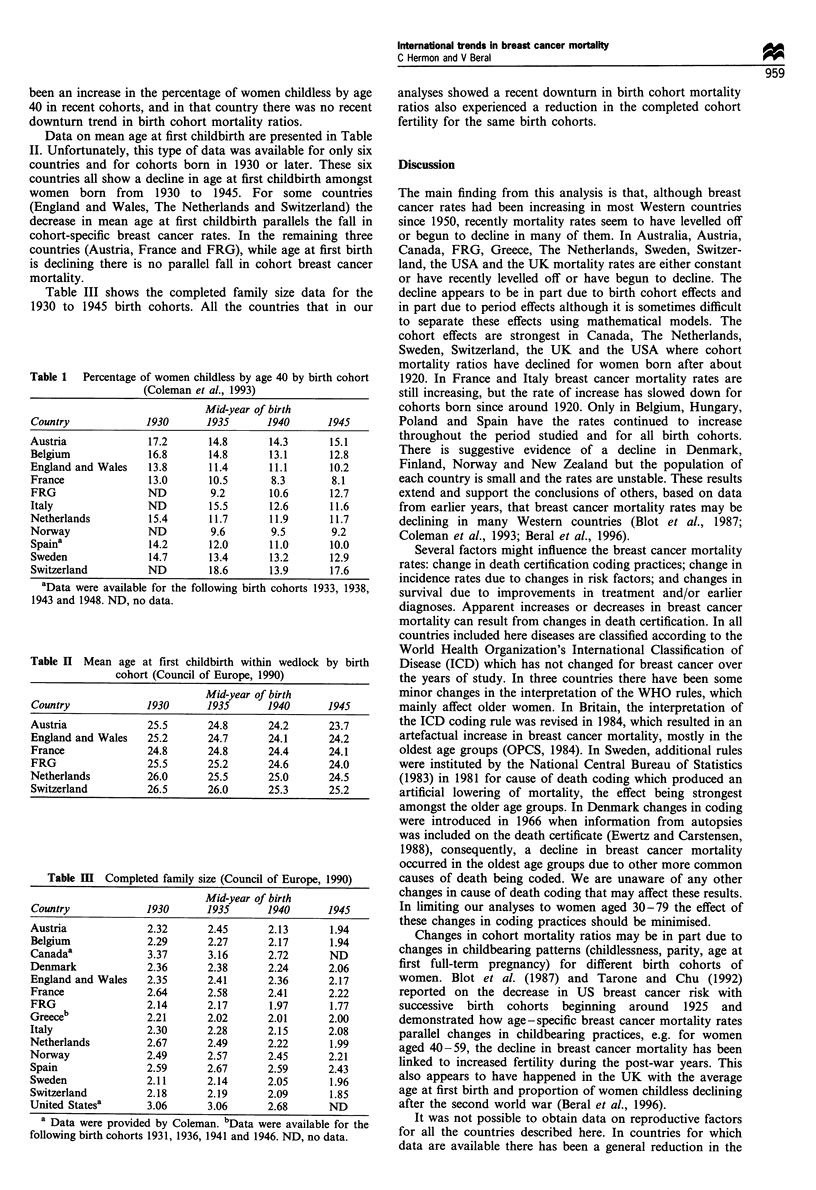

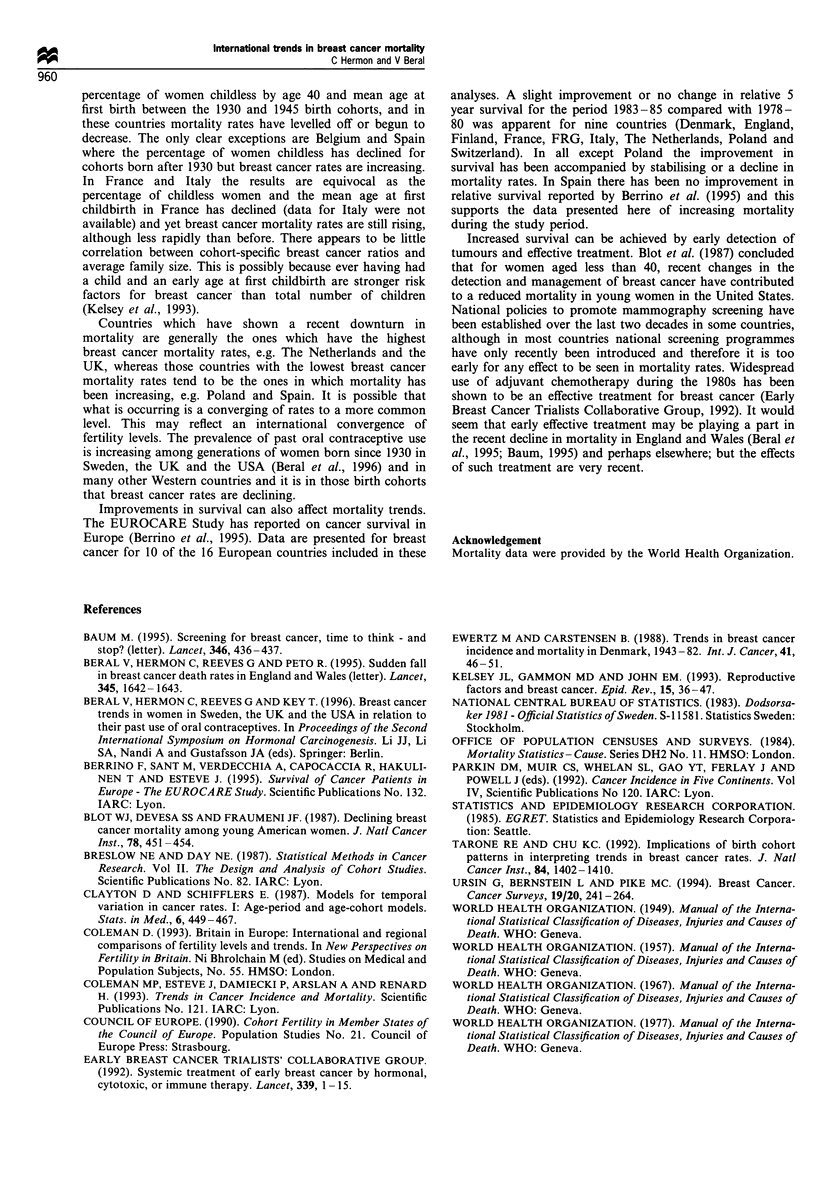


## References

[OCR_00640] Baum M. (1995). Screening for breast cancer, time to think--and stop?. Lancet.

[OCR_00646] Beral V., Hermon C., Reeves G., Peto R. (1995). Sudden fall in breast cancer death rates in England and Wales.. Lancet.

[OCR_00664] Blot W. J., Devesa S. S., Fraumeni J. F. (1987). Declining breast cancer mortality among young American women.. J Natl Cancer Inst.

[OCR_00672] Clayton D., Schifflers E. (1987). Models for temporal variation in cancer rates. I: Age-period and age-cohort models.. Stat Med.

[OCR_00698] Ewertz M., Carstensen B. (1988). Trends in breast cancer incidence and mortality in Denmark, 1943-1982.. Int J Cancer.

[OCR_00705] Kelsey J. L., Gammon M. D., John E. M. (1993). Reproductive factors and breast cancer.. Epidemiol Rev.

[OCR_00725] Tarone R. E., Chu K. C. (1992). Implications of birth cohort patterns in interpreting trends in breast cancer rates.. J Natl Cancer Inst.

[OCR_00730] Ursin G., Bernstein L., Pike M. C. (1994). Breast cancer.. Cancer Surv.

